# Recovery of a *Burkholderia thailandensis*-like isolate from an Australian water source

**DOI:** 10.1186/1471-2180-8-54

**Published:** 2008-04-02

**Authors:** Jay E Gee, Mindy B Glass, Ryan T Novak, Daniel Gal, Mark J Mayo, Arnold G Steigerwalt, Patricia P Wilkins, Bart J Currie

**Affiliations:** 1 Bacterial Zoonoses Branch, Division of Foodborne, Bacterial, and Mycotic Diseases, National Center for Zoonotic, Vector-Borne, and Enteric Diseases, Centers for Disease Control and Prevention, 1600 Clifton Rd., NE., MS-G34, Atlanta, Georgia, 30333, USA; 2 Menzies School of Health Research, Charles Darwin University, Darwin, Northern Territory, Australia

## Abstract

**Background:**

*Burkholderia thailandensis*, a close relative of *Burkholderia pseudomallei*, has previously been reported only from Southeast Asia and North America. It is biochemically differentiated from *B. pseudomallei *by the ability to utilize arabinose. During the course of environmental sampling for *B. pseudomallei *in the Northern Territory of Australia, an isolate, MSMB 43, was recovered that is arabinose positive.

**Results:**

Genetic analysis using 16S rDNA sequencing and DNA/DNA hybridization indicates that MSMB 43 is most similar to *B. thailandensis *although multi-locus sequence typing indicates that this isolate is divergent from both *B. pseudomallei *and other described *B. thailandensis*.

**Conclusion:**

We report the isolation and initial characterization of strain MSMB 43, which is a *B. thailandensis*-like isolate recovered in Australia.

## Background

*Burkholderia thailandensis *is a less virulent close relative of *Burkholderia pseudomallei*, the causative agent of melioidosis [1]. *B. thailandensis *can be differentiated biochemically from *B. pseudomallei *by its ability to assimilate arabinose [1,2]. During the course of environmental surveys for *B. pseudomallei *in the rural region outside Darwin, Northern Territory, Australia, an arabinose assimilating isolate, designated strain MSMB 43, was recovered from a bore water source that also yielded *B. pseudomallei *from the same and subsequent samples.

We report the initial characterization of a *B. thailandensis*-like isolate, the first of its kind, in Australia. In this study we characterize strain MSMB 43 using phenotypic tests, 16S rDNA gene sequencing, multi-locus sequence typing (MLST), and DNA-DNA hybridization.

## Results and Discussion

Standard biochemical testing, including arabinose assimilation, identified strain MSMB 43 as *B. thailandensis*. However, MSMB 43 did not grow at 42°C and produced little to no gas from nitrate [1,3]. A *B. pseudomallei*-specific real-time PCR method targeting a gene in the type III secretion system (TTS) was performed as previously described. Strain MSMB 43 was negative by TTS real-time PCR suggesting that it was not a *B. pseudomallei *[4].

The accession numbers for the 16S rDNA sequences determined for strains MSMB 43 and *Burkholderia thailandensis *ATCC 700388^T ^are [GenBank: EF114404] and [GenBank: EF535235] respectively. The 16S rDNA sequence of strain MSMB 43 has 99.7% similarity and 98.9% identity to the 16S rDNA sequence of the *B. thailandensis *type strain. The discrepancy between the % similarity and % identity is due to multiple heterogeneous base calls in the sequence for MSMB 43, probably resulting from differences in alleles of the 16S rDNA, which has been previously noted for *B. pseudomallei*, but not in *B. thailandensis *[1,5].

**Figure 1:**
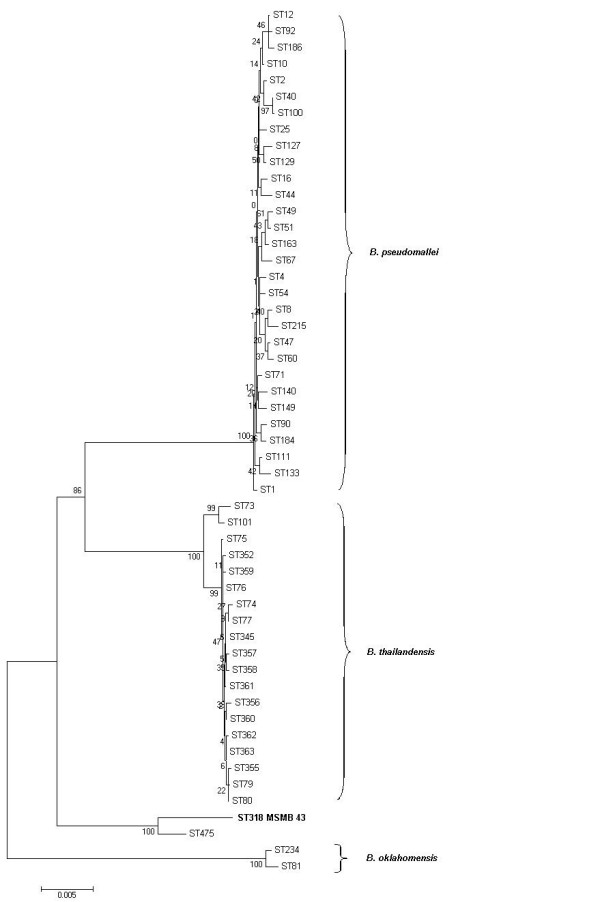
**MEGA 3.1 analysis of concatenated MLST sequences.** Strain MSMB 43 – ST318 is compared to a selected subset of STs from the mlst.net database using Neighbor-joining Kimura 2-parameter with 1000 step bootstrap.

To characterize MSMB 43 further, we performed multi-locus sequence typing (MLST), which uses the DNA sequencing of defined segments of seven housekeeping genes to determine strain relatedness [6]. Strain MSMB 43 was assigned a sequence type (ST) of 318 on the MLST website. None of the alleles for strain MSMB 43 matched alleles of *B. thailandensis *strains available in the MLST database [7]. A dendrogram using concatenated sequences of all *B. thailandensis *STs in the database, along with a representative set of *B. pseudomallei *and *B. oklahomensis *sequences, indicates that ST 318 of strain MSMB 43 does not cluster with any STs except for ST475 (Fig. 1). ST475 is represented by only one example, strain 1554, which is an unassigned *Burkholderia *species from a water sample from another bore in the rural Darwin region that had also yielded *B. pseudomallei *isolates. MSMB 43 and strain 1554 share one allele, *narK*, by MLST. Comparing the concatenated sequences directly indicates that the sequence for ST 318 has an identity of 96.8%, 96.3%, and 95.6%, respectively, to the sequences of the type strains of *B. thailandensis*,  *B. pseudomallei*, and *B. oklahomensis*.

To determine the relatedness of strain MSMB 43 to the *B. thailandensis *type strain at the whole genome level DNA-DNA hybridization was performed as previously described [8,9]. A 91% level of relatedness was determined when DNA from strain MSMB 43 was labeled and in the reciprocal test using labeled DNA from the *B. thailandensis *type strain (Table 1).

**Table 1:** DNA-DNA hybridization using labeled DNA from MSMB 43 and from the *Burkholderia thailandensis *type strain.

Source of unlabeled DNA	Results with labeled DNA from *B. thailandensis *ATCC 700388^T^		Results with labeled DNA from strain MSMB 43	
	RBR^a ^@65°C	D^b^	RBR^a ^@65°C	D^b^

*B. thailandensis *ATCC 700388^T^	100	0.0	91	0.4
MSMB 43	91	4.0	100	0.0

^a^Relative Binding Ratio: the amount of double-stranded DNA formed between labeled and unlabeled DNAs from different strains divided by the amount of double-stranded DNA formed between labeled and unlabeled DNA from the same strain. RBR is expressed as a percentage.

^b^Divergence within related sequences calculated on the assumption that each 1°C decrease in the thermal stability of a DNA duplex is caused by 1% of unpaired bases within that duplex. D was calculated to the nearest 0.5%.

*Burkholderia pseudomallei *is a major cause of community acquired septicemia in Thailand and in tropical northern Australia, especially the Darwin region of the Northern Territory [10,11]. As an environmental saprophyte, it has a wide distribution in endemic areas and poses a hazard for those who are exposed to it. In northern Australia, the ability to distinguish the pathogen *B. pseudomallei *from less or non-pathogenic species is important since public health action may result [4,5,9,10]. For instance, the misidentification in the U.S. of *B. thailandensis *as *B. pseudomallei *could potentially trigger an unnecessary bioterrorism investigation.

Strain MSMB 43 appears distinct from *B. thailandensis*, *B. pseudomallei*, and *B. oklahomensis *by analysis of MLST sequences, but unexpectedly the percent DNA-DNA hybridization is above the 70% threshold that fulfills part of the current gold standard for species definition [12].

*B. thailandensis *is a close relative of *B. pseudomallei*, but is much less virulent in animal models such as hamsters [1,2]. Since access to *B. pseudomallei *is restricted due to its classification as a category B select agent, *B. thailandensis *and other less virulent related strains may serve as acceptable substitutes for training and research [13,14]. *B. thailandensis *has also been the subject of increased investigation recently, especially in genome sequence comparison studies, to reveal virulence factors in *B. pseudomallei *[14-16].

## Conclusion

We report the isolation and characterization of strain MSMB 43 from bore water in the rural Darwin region of tropical northern Australia. This is the first report of a *B. thailandensis*-like isolate in Australia. Further genomic characterization of MSMB 43 may yield insights into the phylogenetic relatedness of *B. thailandensis *strains and may allow identification of yet undescribed virulence factors in *B. pseudomallei*. The relationship of this isolate to *B. thailandensis *strains from southeast Asia and to endemic Australian *Burkholderia* species requires further elucidation. The genome of MSMB 43 is currently being fully sequenced along with a collection of *B. pseudomallei*, *B. mallei *and *B. thailandensis *strains from various locations [17]. The sequencing project should help unravel the phylogeny of these *Burkholderia *and ascertain if MSMB 43 represents environmental *Burkholderia *species in Australia which are ancestral to both Southeast Asian *B. thailandensis *and *B. pseudomallei*. Recent data show that Australian *B. pseudomallei *strains are probably ancestral to those from southeast Asia [18,19]. The origins of *B. pseudomallei *may therefore be linked to environmental *Burkholderia *species in Australia such as MSMB 43. In addition, comparison of *B. pseudomallei *and closely related *Burkholderia *strains collected concurrently from environmental sources may provide further insights into horizontal gene transfer among these species [14].

## Methods

### Biochemical testing

Standard biochemical testing was performed as outlined in Weyant *et al *[3]. Arabinose assimilation was tested using a minimal salts solution with 10% L-arabinose.

### DNA preparation

Whole cell suspensions of bacteria were used for this study as previously described [5]. Bacteria were grown by plating one loop (1 μl) of stock cell suspension in defibrinated rabbit blood (stored at -70°C until use) on trypticase soy agar with 5% defibrinated sheep blood (SBA) (BBL Microbiology systems, Cockeysville, MD) and incubated aerobically 1–2 days at 37°C. A single colony was suspended in 200 μl of 10 mM Tris, pH 8.0 in a 1.5 ml Millipore 0.22 μm filter unit (Millipore, Bedford, MA), heated at 95°C for 30 min, and centrifuged at 6000 × g for 5 min.

### 16S rDNA sequencing

Sequencing and analysis of 1488 bp of the 16S rDNA was performed as previously described [5]. In brief, we used the Expand Hi Fidelity PCR system (Roche, Indianopolis, IN). The amplification mix consisted of 1× buffer #2, 200 μM dNTP mix, 0.4 μM primers F229 and R1908, 5 units Expand Polymerase, and 2 μl of cell extract in a total volume of 50 ul. Reactions were first incubated for 5 min at 95°C. Then, 35 cycles were performed as follows: 15 sec at 94°C, 15 sec at 60°C, and 1 min and 30 sec at 72°C. Reactions were then incubated at 72°C for an additional 5 min. PCR products were purified with Qiaquick PCR purification kit (Qiagen, Valencia, CA).

Sequencing primers were chosen from a panel of previously described oligonucleotides [5]. Sequencing was performed using an Applied Biosystems (ABI) BigDye terminator cycle sequencing ver 3.1 kit as per the manufacturer's instructions, except 0.25 μl of BigDye 3.1 were used for each reaction instead of 8 μl (Applied BioSystems, Foster City, CA). Sequencing products were purified by using Centri-Sep spin columns (Princeton Separations, Adelphia, NJ) and were resolved using an Applied Biosystems model 3130xl automated DNA sequencing system (Applied BioSystems, Foster City, CA). BestFit in the Wisconsin package (Accelrys, San Diego) was used to assess the percent identity between sequences.

### Multi-locus sequence typing

We used the panel of primers described on the MLST Web site and in Godoy *et al*. for both amplification and sequencing [6,7]. For amplification, we used the Expand Hi Fidelity PCR system. Amplification mix consisted of 1× buffer #2, 200 μM dNTP mix, 0.4 μM forward and reverse primers, 0.9 units Expand Polymerase, and 1 μl of cell extract in a total volume of 25 ul. Conditions consisted of an initial 5 min hold at 95°C, followed by 35 cycles at 95°C for 30 sec, 60°C for 30 sec, and 72°C for 1 min with a final hold at 72°C for 10 min. PCR products were purified and sequenced as described above for the 16S rDNA sequencing except using the MLST primer set.

To determine relatedness to other *Burkholderia *strains, the sequences of the seven alleles were concatenated and analyzed using the Neighbor-joining, Kimura 2-parameter method with 1000 step bootstrap in MEGA 3.1 [6,20]. BestFit was used to assess the percent identity between concatenated sequences.

### DNA/DNA hybridization

DNA-DNA hybridization was performed using MSMB 43 and the type strain of *B. thailandensis*, strain ATCC 700388^T^. Cells were harvested and lysed, and the chromosomal DNA was isolated and purified as previously described [8]. DNA from strain MSMB 43 and strain ATCC 700388^T ^were labeled with [^32^P]dCTP using a commercial nick translation kit (Invitrogen Life Technologies, Carlsbad, CA) and tested for reassociation to unlabeled DNA from the same strain (homologous reaction), as well as to each other (heterologous reactions). Relative binding ratios and percent divergence were calculated as described previously [8].

## Authors' contributions

JEG carried out the 16S rDNA sequencing, performed analysis of both 16S rDNA and MLST, participated in the design of the study, and drafted the manuscript. MBG, DG, and MJM performed biochemical and phenotypic studies. RTN performed MLST and sequence alignment. AS performed DNA/DNA hybridization. PPW and BJC participated in the design and coordination of the study. The laboratory of BJC and MJM retrieved and cultured the bore water isolates. All authors read and approved the final manuscript.
